# Selective Attention Modulates Human Auditory Brainstem Responses: Relative Contributions of Frequency and Spatial Cues

**DOI:** 10.1371/journal.pone.0085442

**Published:** 2014-01-15

**Authors:** Alexandre Lehmann, Marc Schönwiesner

**Affiliations:** 1 Laboratory for Brain, Music and Sound Research (BRAMS), Montreal, Canada; 2 Department of Psychology, University of Montreal, Montreal, Canada; 3 Centre for Research on Brain, Language and Music (CRBLM), Montreal, Canada; 4 Montreal Neurological Institute, McGill University, Montreal, Canada; Harvard Medical School/Massachusetts General Hospital, United States of America

## Abstract

Selective attention is the mechanism that allows focusing one’s attention on a particular stimulus while filtering out a range of other stimuli, for instance, on a single conversation in a noisy room. Attending to one sound source rather than another changes activity in the human auditory cortex, but it is unclear whether attention to different acoustic features, such as voice pitch and speaker location, modulates subcortical activity. Studies using a dichotic listening paradigm indicated that auditory brainstem processing may be modulated by the direction of attention. We investigated whether endogenous selective attention to one of two speech signals affects amplitude and phase locking in auditory brainstem responses when the signals were either discriminable by frequency content alone, or by frequency content and spatial location. Frequency-following responses to the speech sounds were significantly modulated in both conditions. The modulation was specific to the task-relevant frequency band. The effect was stronger when both frequency and spatial information were available. Patterns of response were variable between participants, and were correlated with psychophysical discriminability of the stimuli, suggesting that the modulation was biologically relevant. Our results demonstrate that auditory brainstem responses are susceptible to efferent modulation related to behavioral goals. Furthermore they suggest that mechanisms of selective attention actively shape activity at early subcortical processing stages according to task relevance and based on frequency and spatial cues.

## Introduction

Selective attention is the ability to focus cognitive resources on sensory information that is relevant to the current goal or task [1]. An example in the auditory domain is the so-called cocktail party phenomenon [2] – the ability to attend to one of several talkers in a noisy environment. Such ability is made possible because different speakers can be identified by different conjunctions of auditory cues, such as spatial location and frequency content over time. Empirical evidence, both from anatomical and functional studies suggest that spatial and non-spatial auditory features are processed by distinct neural pathways [3]. Although controversial [4], this notion of parallel feature processing in audition is seen as valid, at least for early cortical processing stages [5]. Selective attention is known to modulate activity in early sensory cortices and in the human thalamus [6], [7]. An important theoretical and empirical question concerns the earliest sensory processing stage at which activity is modulated by attention.

The mammalian auditory system contains an extensive network of descending, or efferent, pathways. These efferent fibres terminate at all major levels of the auditory system, including the thalamus, the inferior colliculus, the superior olivary complex, and the cochlear nucleus [8]–[11] and affect many aspects of subcortical neuronal responses, including filtering [12] and sharpness of tuning [13]. The efferent pathways may thus provide a mechanism for an influence of attention and behavioral goals on neural activity at subcortical levels and short time scales. The frequency following response (FFR) may reflect some of the efferent influence of early processing. It is a component of the auditory brainstem response that occurs in response to a periodic stimulus [14], and has been used as a measure of neural phase-locking to the period of the fundamental frequency (F0) of a stimulus in the brainstem. Recent studies have shown experience-dependent plasticity in the FFR [14] and suggested that presentation context or repetition may modulate the FFR [15], [16].

The dichotic listening paradigm, in which different stimuli are presented to each ear, is an often-used approach to assess selective attention to auditory information in an experimental model of a “cocktail party” situation, in which one of two sound streams must be selected and attended to. Studies from Galbraith and colleagues, measuring the FFR in a dichotic listening paradigm, have suggested that selective attention can modify brainstem evoked responses in humans [17]–[20]. In one of those studies [18], participants were presented with speech sounds and spontaneously directed their attention to a particular ear at their own pace. The authors reported larger responses to the fundamental frequency (F0) of an attended vowel compared to an ignored vowel. Using fMRI, Rinne and colleagues [21] reported stronger activation of the inferior colliculus when attending a contralateral stream of tones versus attending an ipsilateral stream. All aforementioned studies have used dichotic presentation of auditory stimuli of different frequencies, thus creating a situation in which both spatial and frequency cues are available to distinguish the two presented streams. To date, it remains unclear whether the attentional effects reported by those studies are based on spatial and/or on frequency selectivity at the brainstem level. Indeed, their results could be accounted for fully by dichotic presentation, given the fact that signals from both ears undergo relatively independent processing at the brainstem level in dichotic conditions. A recent study by Hairston and colleagues [22] showed a decrease in response to a task-irrelevant background tone when subjects performed a duration discrimination task on visual and auditory stimuli. In the auditory condition, both stimuli were presented diotically, suggesting that an effect of attention can be measured using ABR. However it is unclear whether the suppression they claim is general or frequency-specific, because competing stimuli were not presented simultaneously and frequency was not an experimental variable.

The present experiment directly addressed those issues, by investigating whether endogenous selective attention to one of two simultaneously presented streams of vowel sounds affects amplitude and phase locking of auditory brainstem response when the sound streams were either discriminable by frequency content alone, or both by frequency content and spatial location. We hypothesized that selective attention may modulate brainstem responses under both conditions, that is to say even if the streams are not spatially separable. In the latter case, it is conceivable that selective attention may enhance or suppress frequency components of an attended or ignored sound at subcortical levels, given that efferent projections are mostly tonotopically organized [9], [10], [13] and target structures as early as the cochlear nucleus. Indeed all processing levels of the auditory system, from the cochlea to core auditory cortex, show a topographic representation of sound frequency. Specifically we expected FFR spectral power at both stimuli’s F0, an index of pitch processing, to be modulated by selective attention. We predicted a modulatory pattern that reflects an increase in spectral power to the attended vowel’s F0 and a decrease in spectral power to the unattended vowel’s F0, on the basis of previous results in the cortex [23]–[25] and brainstem [18], [21].

## Materials and Methods

We investigated whether endogenous selective attention affects the auditory brainstem frequency-following response (FFR), using a pitch discrimination task, with a 2×2 factorial design (attention × spatial separation) in a constant stimulus paradigm.

### Subjects

Fifteen participants (four males, mean age 26.4±5.6) were recruited for the experiment and provided written informed consent. All had normal or corrected-to-normal vision and had no history of hearing disorder or neurological disease. The experimental procedures conformed to the World Medical Association’s Declaration of Helsinki and were approved by the Research Ethics Committee of the Faculty for Arts and Sciences of the University of Montreal.

### Stimuli and Tasks

Auditory stimuli consisted of two recorded French vowels:/a/uttered by a male speaker and/i/uttered by a female speaker. The fundamental frequency of both recorded vowels was set constant using the STRAIGHT toolbox [26] to create the standard vowel sounds (standard F0 was 170 Hz for the male and 225 Hz for the female vowel). A pitch-shifted version (−4%) of each standard vowel was used as target sounds (163 Hz and 216 Hz for the male and female targets respectively). Sounds were 210 ms in duration with 8 ms cosine rise/fall time, low-pass filtered at 1500 Hz. Synchronous male and female sequences of vowel sounds were presented, with an ISI of 90 ms. Five percent of presented stimuli were targets, each sequence was ordered so that the standard was repeated at least nine times before a target would be presented. According to the spatial separation condition, the presentation of the two sound sequences was either dichotic (male sequence delivered to the left ear and female sequence delivered to the right ear) or diotic (mixed signal of both sequences delivered to both ears). Depending on the attentional condition, subjects were required to attend to the vowels uttered by the designated speaker (attend male or attend female) and detect targets by pressing a button with their right hand.

Relative intensity between female and male vowels was adjusted psychophysically to match subjective loudness in a pilot experiment, as done in a previous study by Galbraith and colleagues [18]. The female voice was always presented 3 dB less than the male voice. Stimuli intensity was measured at the location of the participants’ head with a headphone coupler. During diotic presentation, stimulus intensity was 85 dB SPL at each ear; during dichotic presentation, the female voice was presented at 84 dB SPL and the male voice at 87 dB SPL.

**Figure 1:**
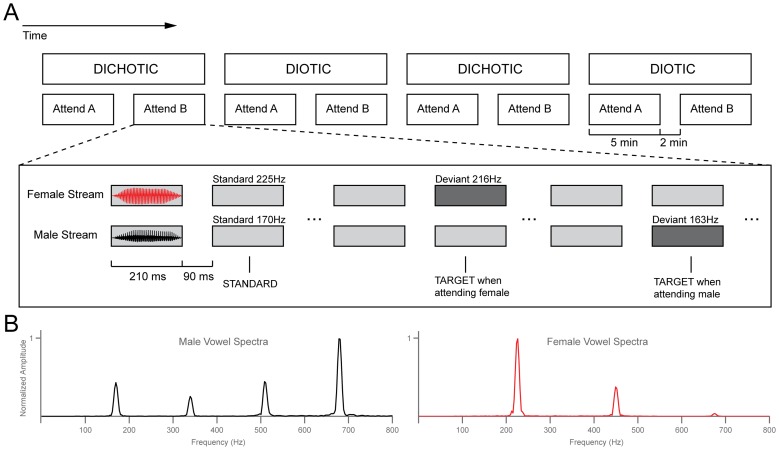
The experimental paradigm. (A) The experiment was composed of eight five-minute blocks. The gender of the voice to be attended in the first block (Attend A) was counterbalanced across subjects, and it alternated in the subsequent blocks. During a given block, male and female standard vowel sounds were presented synchronously every 300 ms and participants had to detect infrequent deviant sounds in the attended voice. Only standard sounds were used in the analysis. (B) shows the spectra of the standard sounds. On the left is the male standard sound with a F0 of 170 Hz and on the right, the female sound with a F0 of 225 Hz.

The sound sequences were presented in five-minute blocks alternating with two-minute breaks. A trial block consisted of a mixture of frequent (n = 950) standard sounds and randomly occurring infrequent (n = 50) target sounds. During the breaks, the experimenter would enter the room, give subjects the opportunity to rest for a minute and instruct them which speaker to attend to during the upcoming block. He would also advise them when the spatial separation condition would change (from dichotic to diotic and from diotic to dichotic). The order of attend male and attend female conditions was randomized. The experiment always started with two dichotic condition blocks. Two blocks of each of the four condition combinations were presented. Figure 1 shows an illustration of the experimental protocol and parameters. Auditory stimuli were delivered via insert earphones (ER3, Etymotic Research, www.etymotic.com). The experiment was performed with Matlab software (The Mathworks, www.mathworks.com) interfaced with a signal processing system (RX6, Tucker-Davis Technologies, www.tdt.com).

### Analysis of Behavioral Data

The sensitivity index (d′) was computed for each participant in each condition to quantify target discriminability. D′ indices were submitted to a 2 (attention)×2 (spatial presentation) analysis of variance (ANOVA). Because of a technical incident, behavioral data from seven participants are available.

### EEG Acquisition and Analysis

Auditory-evoked brainstem potentials were recorded from three sintered Ag/AgCl electrodes that contained the first amplifier stage with the electrode cover (“active electrodes”, BioSemi, www.biosemi.com). Ultra-flat active electrodes were placed at both mastoids and the vertex (Cz). The vertex electrode served as reference for the recording, and two ground electrodes were placed on the central forehead. Active electrodes provide impedance transformation on the electrode to prevent interference currents from generating significant impedance-dependent nuisance voltages. We therefore did not control electrode impedances, but rather kept direct-current offset close to zero during electrode placement. Electrode signals were amplified with a BioSemi ActiveTwo amplifier (BioSemi, www.biosemi.com) with a dedicated bank of hardware amplifiers for low-noise brainstem recordings. The signals were sampled at 16384 Hz, bandpass-filtered online between 100 and 3000 Hz, and stored for offline analysis using BioSemi ActiView software.

The data were processed using the EEGLAB toolbox [27] and in-house developed scripts in Matlab (The Mathworks, www.mathworks.com). Signal was re-referenced to averaged mastoids. It was then segmented into 300-ms epochs ranging from −20 to 280 ms relative to the stimulus onset. The DC offset of each epoch was adjusted so that the average potential of the pre-stimulus baseline was zero. Epochs containing unusually large potentials (outside ±50 µV) were rejected. Further epochs were rejected using EEGLAB’s automatic iterative rejection procedure with an initial threshold of five standard deviations [27]. Both procedures led to the rejection of an average of 6% of epochs. Epochs containing responses to both stimulus polarities were averaged and then summed to optimally measure the frequency-following response [28]. Only responses to the standard sounds were analyzed, yielding an average number of 1784 epochs per condition per participant. To investigate neural phase-locking, the sustained portions (20–195 ms post-stimulus) of the individual FFRs were submitted to a frequency analysis using a zero-padded 4096-point fast Fourier transform with Hanning windows. Normalized mean spectral power in a ten-Hz bin around the standard male and female F0 was computed. To test our hypothesis of increased spectral power to an attended vowel and decreased spectral power to an unattended vowel, group statistics were computed using a 2 (attention)×2 (spatial presentation) analysis of variance (ANOVA) for each vowel. To further probe our hypothesis at the individual level, and estimate the noise level, we computed bootstrap statistics. The standard error of the mean of the spectral power estimates was computed as the standard deviation of a distribution of 10′000 averages of data epochs drawn with replacement from the original data (bootstrap procedure). For a given subject, each average was computed using the total number of available epochs for this subject. In addition, root-mean-square (RMS) power of individual FFRs were computed in the steady-state portion of the FFR for each condition and submitted to the same ANOVA and bootstrap procedure described above, in order to look for possible response amplitude effects.

To investigate cross-subject variations observed in FFR modulation patterns, a third step of analysis was undertaken to capture the overall magnitude of modulatory effects. Specifically for the comparison between dichotic and diotic conditions, we used an index of attentional modulation that is independent of whether the modulation effect is an enhancement or a suppression of response power at male or female F0. This index was computed for each subject as follows:
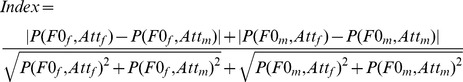
where 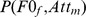 signifies the response power 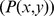 in the frequency band around the female fundamental frequency 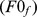 in the condition in which the participant was instructed to attend to the male voice 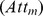, and similarly for the other expressions. Thus absolute differences in response power at either F0 across both attention conditions were normalized by their root-mean-square average.

Correlation between target discriminability and the modulation index were computed using Pearson’s linear coefficient, as well as using robust regression with iteratively reweighted least squares [29], as implemented in Matlab’s corr and robustfit functions respectively.

## Results

### Spectral Power of FFR is Modulated by Attention

**Figure 2:**
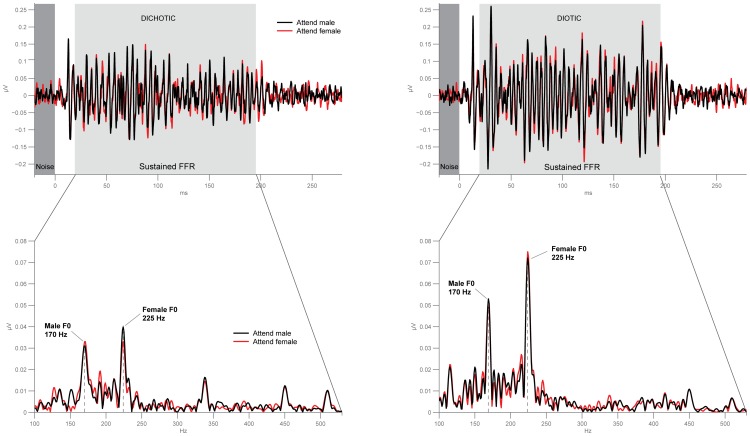
Frequency-following response reflects neural phase-locking to the fundamental frequency of male and female voice. Grand average brainstem frequency-following response (FFR) for each experimental condition, plotted in the time (upper panel) and frequency (lower panel) domain. Red lines indicate the response while attending to the female voice; black lines indicate the response while attending to the male voice. FFR obtained during dichotic presentation are shown on the left, diotic presentation on the right. Both vowels’ fundamental frequencies (170 Hz male, 225 Hz female) yielded clearly identifiable maxima in the individual and average FFR spectra.

**Figure 3:**
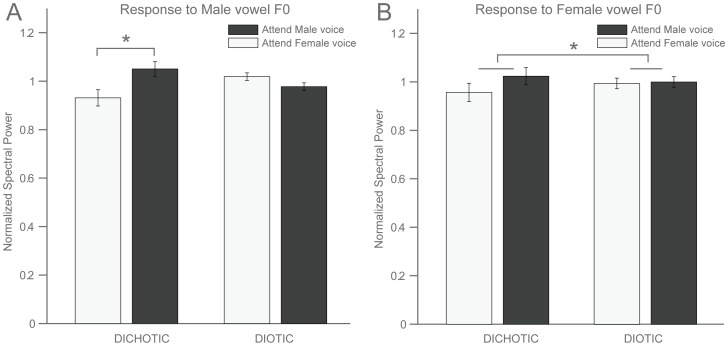
Endogenous selective attention modulates response spectral power around male stimulus’ fundamental frequency. Mean normalized spectral power for each condition, computed around the fundamental frequencies of both the male (A) and the female vowel (B). Error bars indicate the standard error of the mean. (A) In the dichotic condition, spectral power to the male voice was bigger when male voice was attended (p = 0.042). (B) Spatial presentation modulated spectral power to the female voice (p = 0.013).

**Figure 4:**
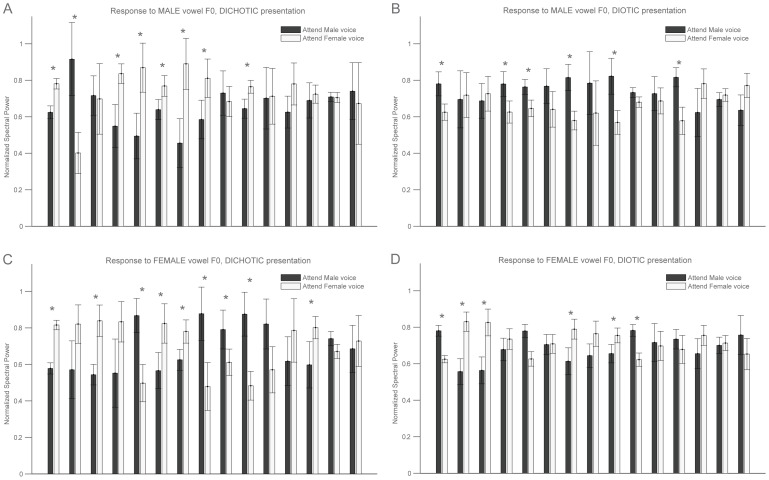
Selective attention modulates brainstem response at individual level, both in dichotic and diotic presentation. Normalized spectral power at the fundamental frequencies of the male (A,B) and female stimuli (C,D) for each participant in the dichotic (A,C) and diotic (B,D) condition. White bars indicate the response amplitude while attending to the female voice; grey bars indicate the response amplitude while attending to the male voice. Error bars indicate the standard error of the mean calculated by bootstrap resampling. Stars indicate a significant difference of two standard errors of the mean (p<0.05) between attending to the male and female voice.

We were able to record sound-evoked potentials from the brainstem with an average signal-to-noise ratio of about 16 dB, (Fig. 2, upper panel). We confirmed that peaks in the spectrum of the recorded responses coincided with peaks in the stimulus spectra (Fig. 2, lower panel). In all experimental conditions, the two largest frequency peaks in the recorded responses corresponded to the fundamental frequencies of the presented male and female vowels. Other peaks corresponded to harmonics of the fundamental frequencies. Normalized spectral power around both male and female fundamental frequencies, as well as RMS power, were submitted to an analysis of variance (ANOVA) with 2 spatial and 2 attentional conditions (Fig. 3). There was a main effect of the spatial condition on the female spectral power (F(1,14) = 8.11, p = 0.013). For the male spectral power, there was a significant interaction between spatial presentation and attention (Spatial×Attention: F(1,14) = 5.48, p = 0.035), namely, in the dichotic condition, the spectral power at the male frequency was bigger when attending male than when attending female (t(14) = 1.87, p = 0.042). This pattern is in agreement with our hypothesis of a directional effect of attention. There was a main effect of spatial presentation on RMS power (F(1,14) = 17.3, p = 0.001), but there were no effects of attention nor interactions between factors. Although there was a significant group effect on neural phase-locking, the direction of modulation varied across listeners. Some participants showed significant enhancement of the representation of the attended sound, others showed significant suppression. Bootstrap statistics were used to analyze changes in the spectral power of the response around the fundamental frequencies of both stimuli for individual participants in each condition (Fig. 4). We observed a significant modulation (p<0.05) of the response power by the direction of attention in ten out of fifteen participants in the dichotic condition and in nine out of fifteen participants in the diotic condition. Assuming the null hypothesis of no effect, the probability of obtaining the reported number of significant results at the given threshold is about 10^−8^. In six out of fifteen participants the direction of attention also modulated the amplitude of the brainstem response (measured as RMS power) when stimuli were presented dichotically. This fraction dropped to three out of fifteen during the diotic stimulus presentation.

### Effect of Spatial Separability

**Figure 5:**
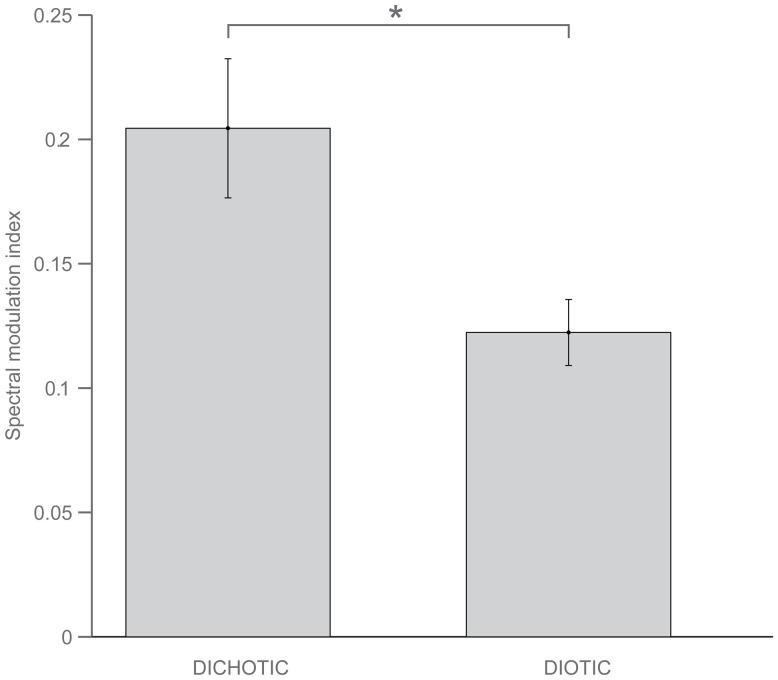
Both spatial and frequency cues contribute to attentional modulation of brainstem response. The average normalized modulation index across participants is significantly higher in the dichotic condition than in the diotic condition (p = 0.006). Error bars indicate the standard error of the mean.

Because some participants showed a significant enhancement of response power of attended stimuli, while others showed a significant suppression, we computed a normalized attentional modulation index that aggregates individual differences in response power, irrespective of their direction, for the purpose of directly comparing the magnitude of effects in dichotic versus diotic presentation. An analysis of variance of the individual modulation indices for the dichotic and diotic conditions revealed a significantly greater modulation when sounds were separable in frequency and across ears than when they were separable only in the frequency domain (F(1,14) = 10.2, p = 0.006, Fig. 5).

### Behavioral Results

**Figure 6:**
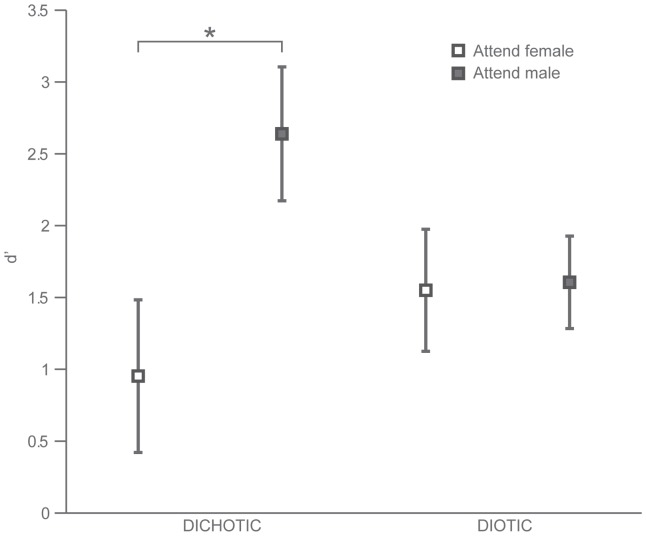
Spatial presentation modulates behavioural discriminability. Average behavioral discriminability of the stimulus streams, expressed as d′ in each condition. The difference between attending to the male versus the female voice is significant in the dichotic condition (p = 0.001), but not in the diotic condition. Error bars indicate the standard error of the mean.

Participants effectively paid attention to the indicated stream of vowels as revealed by an average d′ of 1.69 (hit rate = 85%). There was a main effect of attention on performance (F(1,6) = 11.7, p = 0.0141), as well as an interaction between attention and spatial presentation (F(1,6) = 21.8, p = 0.00345). It was easier for participants to attend the male stream than the female stream in the dichotic condition (t(6) = 5.1973, p = 0.001). There was no performance difference between attending male and female stimuli in the diotic condition (Fig. 6).

### Correlation between Behaviour and Brainstem Responses

**Figure 7:**
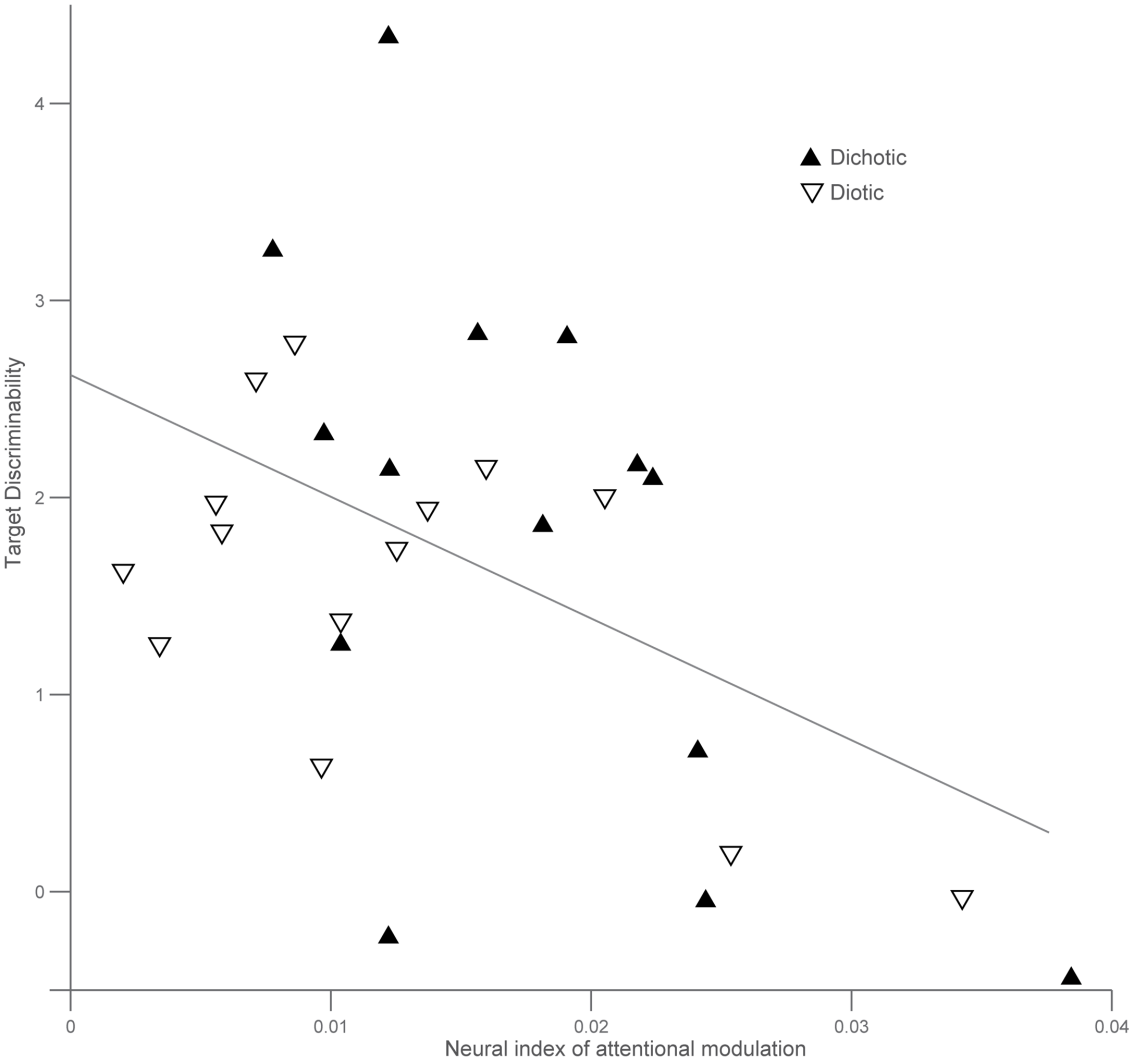
Attentional modulation of the brainstem response correlates with behaviour. The individual neural attentional modulation indices and target discriminability measures (d′) are negatively correlated in both the dichotic and diotic condition (respectively r = −.56, p = 0.037 and r = −.58, p = 0.031). Each triangle represents data from one participant in either the dichotic (solid triangle) or the diotic (hollow triangle) condition. Participants correspond to numbers 1, 5, 7, 9, 12, 13 and 14 as shown on Fig. 4.

Given the observed individual variations in modulation patterns, we examined whether individual differences in task performance account partly for the observed variance in brainstem responses. Correlation statistics between neural modulation index and d′ were performed separately for each condition and were plotted together on Fig. 7. In both the dichotic and diotic condition, the individual attentional modulation index showed a significant negative correlation with individual perceptual discriminability of the stimulus streams (r = −.56, p = 0.037 for dichotic; r = −.58, p = 0.031 for diotic). To assess whether potential outliers could bias the reported correlation, we performed robust regression statistical analysis. Correlation results were replicated using robust regression (p = 0.04 and p = 0.035 for dichotic and diotic respectively).

## Discussion

The goal of the present experiment was to investigate whether selective attention to different acoustic features, such as voice pitch and speaker location, in an active detection task, modulates the auditory brainstem frequency-following response in humans. Specifically, we hypothesized that FFR spectral power would increase for attended vowels, and that it would be the case for both dichotic and diotic presentation.

### Brainstem Responses are Modulated by Selective Attention

We found that endogenous selective attention modulated human brainstem responses when stimuli were discriminable based on spatial and frequency features but also based on frequency features alone (Fig. 3 and 4). The average modulation observed in the most favorable case, i.e. when stimuli were presented to different ears, was about 13%, corresponding approximately to a 10 dB increase in signal amplitude (estimated from table showing wave V amplitude as a function of stimulation loudness in [30]). These results are in agreement with previous findings that endogenous attention can modulate brainstem responses [18], [21], [22]. The attentional modulation was specific to the voice pitches of the competing stimulus streams in that it was observed at the frequency bands corresponding to their fundamental frequencies. In addition, there was a less specific increase in overall response amplitude in a smaller proportion of participants.

### Attentional Modulation Operates in Absence of Spatial Separability

We found that selective attention modulated brainstem responses in the majority of participants when stimulus streams were presented diotically and thus separable only by frequency content. This result demonstrates that selective attention modulates the FFR even in the absence of spatial separability between the two stimuli. This is, to our knowledge, the first evidence for a frequency-specific effect of selective attention on auditory subcortical structures. Such an effect has been shown at the cortical level using frequency tagging of auditory objects [25], [31]. The difference in modulation magnitude observed with and without spatial separability demonstrated that spatial separability contributed approximately 40% over the modulation due to selective attention in the present experiment, and probably in similar proportion to previous studies’ claims. Whereas attentional modulation in the case of spatially separable sounds can be accounted for by the fact that dichotic signals undergo relatively independent processing at the brainstem level, the modulation by attention to frequency features alone suggests the existence of an early neural attention mechanism, likely mediated by efferent input to brainstem structures, that selectively enhances or suppresses the representation of frequency components of sounds depending on task relevance.

### Individual Differences in Attentional Modulation of Brainstem Response Correlate with Behavior

By using bootstrap statistics we observed that the direction of the modulation differed across listeners, some participants showed significant enhancement of the representation of the attended sound, other showed significant suppression. Since results from most previous studies are reported at the group level, it is difficult to know whether their paradigm yielded consistent directional effects at the individual level, or whether, as is the case here, the observed global trend is the net result of multi-directional individual effects. Using a non-directional index, we observed that neural attentional modulation was negatively correlated with behavioral discriminability of the streams. The fact that there is a consistent relationship between neural modulation and behavioral performance suggests a biological relevance of these differences. Participants who struggled with the task (low d′) and presumably expended greater effort on attending selectively, showed greater modulation of brainstem potentials. On the contrary, participants whose performance was at ceiling level showed less modulation.

Another way to interpret individual differences in online attentional modulation relates to experience-dependent plasticity. Brainstem FFR differs for populations of listeners with different histories of sound exposure and expertise, such as musicians and bilinguals, but also persons with autism spectrum disorders or dyslexia. Life-long experience and exposure to specific sound patterns appears to generate plastic changes in the auditory brainstem [14], [32]–[34]. Whether those changes affect brainstem structures directly or via efferent pathways is unknown. The pattern of online modulation of brainstem responses by endogenous attention could thus differ between individuals according to their lifelong expertise or stimulus history.

### Limitations and Perspectives

Given that the experiment was conducted in a bilingual city and at an institute for music research, participants had a variety of levels of language and music expertise, which might at least partly underlie the observed inter-individual variations [33], [35]. We did not control for these factors in the present experiment. Musical and language experience were collected retrospectively from seven participants. Formal musical training varied from zero to eighteen years and participants reported speaking two to five languages, confirming the heterogeneity of the group.

Results at the group level for the male spectral power using dichotic presentation are consistent with the results of Galbraith and colleagues [18], who reported that spectral power increased in response to the attended vowel. However there was no significant effect on the group level for the female spectral power. This may be due to a limitation of the present study, in that the male vowel was always presented to the left ear during dichotic listening. There is evidence for a very small but measurable ear-preference effect when listening to speech stimuli [36], which could contribute to the assymetry observed for the male and female vowels. The nature of the task employed can also explain greater variability in our data. We used an active task that potentially invites a variety of behavioral strategies, such as focusing on the target stimulus stream, or actively suppressing the interfering stream. In an earlier study by Galbraith and colleagues [17] using active tasks, FFR spectral power to the attended stimulus was reported to either increase or decrease depending on the stimulus type and task difficulty. The task employed in the present experiment was relatively easy (average accuracy was 85%). It has been reported [6], [17] that difficult behavioral tasks are required to evoke measurable modulations of early auditory activity. Given the observed relationship between target discriminability and neural modulation in our paradigm, the use of a more difficult task might increase the extent to which brainstem activity is modulated on-line. In follow-up experiments, it would likely be important to use a demanding task that requires substantial attentional effort, while controlling for alternative strategies. Simultaneous acquisition of cortical responses is a possible venue to assess individual strategies.

Similarly to Hairston and colleagues [22], we correlated individual neural modulations of the FFR with sensory performance metrics. This approach aides in illustrating differences inside a population which might otherwise not be captured by group analysis. For instance, Skoe and colleagues (2013) recorded ABR while participants listened to random or patterned sound sequences in a statistical-learning paradigm. As in our study, the direction of the change in ABR across conditions was inconsistent, but the individual changes in ABR correlated with learning performance.

From our data, it is impossible to determine the precise brainstem structures that contribute to the attentional modulation, because the FFR is a sustained potential and does not contain timing information that can be tied to different brainstem nuclei. Such information would be available in click-evoked responses. In non-human animals, studies have reported effects of attention on click-evoked responses in the inferior colliculus in rats [37] and at the cochlear level in rats [38], cats [39] and chinchillas [40]. So far there is no evidence, that click-evoked brainstem responses in humans are modulated by attention [41], [42]. Combining the present approach with fMRI or with intracranial recordings may allow further specification of the locus of the modulation.

Finally, we report here the contribution of frequency cues, but we cannot with our study distinguish whether the modulation is based on place or rate code of frequency information. Indeed such information may be extracted from spectral peaks, or temporal fine-structure (periodicity) cues. A mechanism which targets portion of the tonotopic maps in brainstem structures is easier to imagine than one that targets repetition rates, and we are currently devising an experiment to separate temporal and spectral cues to frequency information.

## Conclusion

In summary, we demonstrated that selective attention modulates the human brainstem frequency-following response, based on frequency cues alones. Modulation was specific to the frequency band of relevance for the task at hand. Isolating this frequency-specific effect made possible an estimation of the relative contribution of spatial and frequency information. Individual neural modulation indices correlated with psychophysical discriminability of the stimuli, suggesting that the modulation was biologically relevant. Our findings demonstrate that auditory brainstem responses are susceptible to efferent modulation related to behavioral goals and suggest the existence of an early neural attention mechanism, which selectively enhances or suppresses stimulus representations in the brainstem based on spatial and frequency cues. Although it is beyond the scope of the present study, we speculate that attentional modulation of brainstem activity is likely mediated by the numerous efferent projections from the cortex to auditory brainstem structures [8]–[11]. Efferent projections can change neural function on a short time-scale; portions of the connections follow the tonotopic organization of the afferent system and may thus modulate responses in a frequency-specific manner. The results support the view that many, if not all, aspects of auditory perception are the result of a dynamic interplay between structures at different hierarchical levels [15], [43]–[45], rather than purely feedforward processing from lower to higher levels.
